# Welcoming the JMA Journal’s Call for Manuscripts on Medical Artificial Intelligence

**DOI:** 10.31662/jmaj.2024-0147

**Published:** 2024-09-06

**Authors:** Shigeki Matsubara

**Affiliations:** 1 Department of Obstetrics and Gynecology, Jichi Medical University, Tochigi, Japan; 2 Department of Obstetrics and Gynecology, Koga Red Cross Hospital, Koga, Japan; 3 Medical Examination Center, Ibaraki Western Medical Center, Chikusei, Japan

**Keywords:** academic writing, artificial intelligence, image analysis, paper, paper writing

## Dear Editors,

Professor Mori, an esteemed physician–researcher, highlighted the promising aspects of artificial intelligence (AI) in medicine and its drawbacks ^(1)^, providing important suggestions to overcome the latter. He recommends submitting manuscripts on medical AI to the JMA Journal. I welcome his statements. Please permit my small voicing.

First, discussions on medical AI should be divided into writing versus other than writing ^(2)^. Professor Mori mainly focused on AI for image analysis and prediction models. Not all researchers will attempt to “develop” AI-based models, whereas all researchers and authors must “write.” Although I have written 548 PubMed-indexed papers, I, a non-statistician, depended on statistics specialists. I never consider a non-specialist should ignore areas outside one’s specialty. I studied and understood statistics “fundamentals.” The same holds true for AI-based research. If some researchers have devised accurate usable AI-based technologies, others will employ suitable ones for their study with “fundamental” knowledge of the corresponding AI and then write papers. Thus, the number of readers interested in AI-based writing may surpass, at least be equal to, those considering AI for other applications. AI-based writing will attract many journal readers, and thus, I hope this issue will be widely discussed in the JMA Journal.

Second, regarding the regulation of AI use, Professor Mori concluded his paper by stating “when submitting, read the Use of Artificial Intelligence-Assisted Tools/Technologies” in the JMA Journal. It indicates: i) AI cannot be an author, ii) authors should declare AI use, and iii) authors should be responsible for the product. These three concepts are reasonable but vague for practical use. Some may even consider, “One can use AI as much as one wishes only if one adheres to these three.” A new standardized guideline is reportedly under construction ^(3)^. I hope that the JMA Journal regulation will be modified to meet the forthcoming worldwide guidelines and will become more practical.

Third, I wish authors of medical AI articles to express their fundamental stance (agree/disagree) on AI in their corresponding topics. Professor Mori clearly states his stance, fundamentally agreeing with AI use (other than writing) and making suggestions to overcome drawbacks. I claimed that, in writing, AI should be used only as a linguistic checker, fundamentally disagreeing with AI use in writing ^(2), (4)^. Depending on the situation, one remains “neutral”: authors should state their stances, including “neutral,” which makes the paper context clearer. This mimics, “state conclusion,” paper-writing fundamentals ^(5)^.

I look forward to reading medical AI articles in the JMA Journal. We have just opened the door to a new scientific world.

## Article Information

### Conflicts of Interest

None

### Author Contributions

S. Matsubara: Identification of the significance and manuscript writing.

### Approval by Institutional Review Board (IRB)

Not applicable

### Data Availability

Data sharing is not applicable to this article, as no new data were created or analyzed in this study.

### Patient Anonymity

Not applicable

### Informed Consent

Not applicable
